# Identification of novel candidate biomarkers for pancreatic adenocarcinoma based on TCGA cohort

**DOI:** 10.18632/aging.202494

**Published:** 2021-02-11

**Authors:** Yang Jie, Wang Peng, Yuan-Yuan Li

**Affiliations:** 1 Department of Pharmacy, Shandong Provincial Hospital, Jinan 250022, Shandong, P.R. China; 2 Department of Pharmacy, The First Affiliated Hospital of Shandong First Medical University, Jinan 250014, Shandong, P.R. China

**Keywords:** pancreatic adenocarcinoma (PAAD), biomarker, weighted co-expression network analysis (WGCNA), connectivity map (CMap), small molecule drug

## Abstract

Pancreatic adenocarcinoma (PAAD) is the most serious solid tumor type throughout the world. The present study aimed to identify novel biomarkers and potential efficacious small drugs in PAAD using integrated bioinformatics analyses. A total of 4777 differentially expressed genes (DEGs) were filtered, 2536 upregulated DEGs and 2241 downregulated DEGs. Weighted gene co-expression network analysis was then used and identified 12 modules, of which, blue module with the most significant enrichment result was selected. KEGG and GO enrichment analyses showed that all DEGs of blue module were enriched in EMT and PI3K/Akt pathway. Three hub genes (*ITGB1*, *ITGB5*, and *OSMR*) were determined as key genes with higher expression levels, significant prognostic value and excellent diagnostic efficiency for PAAD. Additionally, some small molecule drugs that possess the potential to treat PAAD were screened out, including thapsigargin (TG). Functional *in vitro* experiments revealed that TG repressed cell viability via inactivating the PI3K/Akt pathway in PAAD cells. Totally, our findings identified three key genes implicated in PAAD and screened out several potential small drugs to treat PAAD.

## INTRODUCTION

Pancreatic adenocarcinoma (PAAD) is the seventh most common malignancy worldwide [1]. Statistical data suggest that there were about 458, 918 new cases and 432, 242 deaths caused by PAAD in 2018, accounting for 2.5 % of all new cases and 4.5 % of total deaths. It is estimated that PAAD will become a higher ranking owing to the increasing morbidity and mortality [2]. PAAD is characterized by the propensities of untimely diagnosis, easy metastasis, and resistance to current chemotherapeutic treatments [3], and thereby, PAAD has a low five-year survival rate, less than 10 % [4]. Therefore, it is of great importance to explain the potential molecular mechanisms implicated in the development of PAAD, and identify promising biomarkers and novel therapeutic drugs.

PAAD is one of the most serious solid tumors with high complexity in human [5]. The regulation of molecular mechanisms in tumorigenesis has been regarded as the most vital factor associated with PAAD. Recently, on the basis of big integrated data and bioinformatics analysis, the key genes related with cancer development and prognosis could be identified with the widespread popularity of gene chips and the rapid development of high-throughput sequencing technology. Potential hub genes linked with the pathogenesis of PAAD were screened out using bioinformatics meta-analysis based on the GEO and TCGA databases [1]. However, plenty DEGs were obtained after differential gene analysis. Weighted gene co-expression network analysis (WGCNA) is proposed as a systematic biological methodology which could be utilized to construct the co-expression networks of key genes and inquire the association of different sets of genes, within sets of genes and clinicopathological characteristics. It provides an effective functional interpretation tool for identification of candidate key genes, sheds novel insights for cancer treatments, and has been employed in multiple cancers, such as stomach adenocarcinoma [6], clear cell renal cell carcinoma [7], and non-small-cell lung cancer [8]. Furthermore, five hub miRNAs have been identified as potential diagnostic and prognostic candidates for pancreatic ductal adenocarcinoma [9]. Herein, we established a co-expression network using WGCNA on the basis of TCGA and Oncomine databases, and identified three hub genes in PAAD. KEGG and GO enrichment analyses were performed to further explore the functional roles of three hub genes. Overall survival was detected using Kaplan-Meier method and small molecule drugs that have potential to treat PAAD were screened out. Data form the Oncomine dataset were applied as a valuation cohort.

## RESULTS

### Identification of DEGs in PAAD

**Figure 1:**
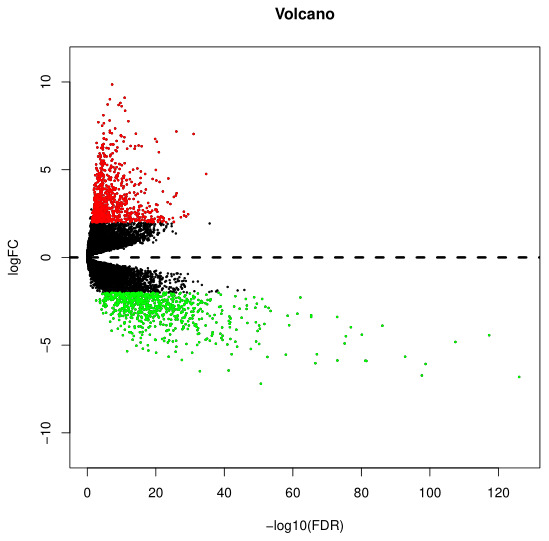
**Identification of DEGs in PAAD based on the TCGA and GTEx datasets.** Volcano plot of gene expression profile data between PAAD and normal samples. Red dots represent upregulated genes in PAAD; green dots represent down-regulated genes; black dots represent non-DEGs. |logFC|>1, FDR<0.05. DEGs, differentially expression genes; PAAD, pancreatic adenocarcinoma; TCGA, the Cancer Genome Atlas; GTEx, Genotype-Tissue Expression.

After integrated bioinformatics analyses for TCGA-PAAD and GTEx datasets, a total of 4, 777 protein-coding genes were found to be differentially expressed with the threshold of |logFC| > 1 and FDR < 0.05. The volcano plot of DEGs in PAAD was presented in Figure 1. Among these DEGs, 2, 536 DEGs were up-regulated and 2, 241 DEGs were down-regulated.

### Weighted co-expression network establishment and key modules identification

**Figure 2:**
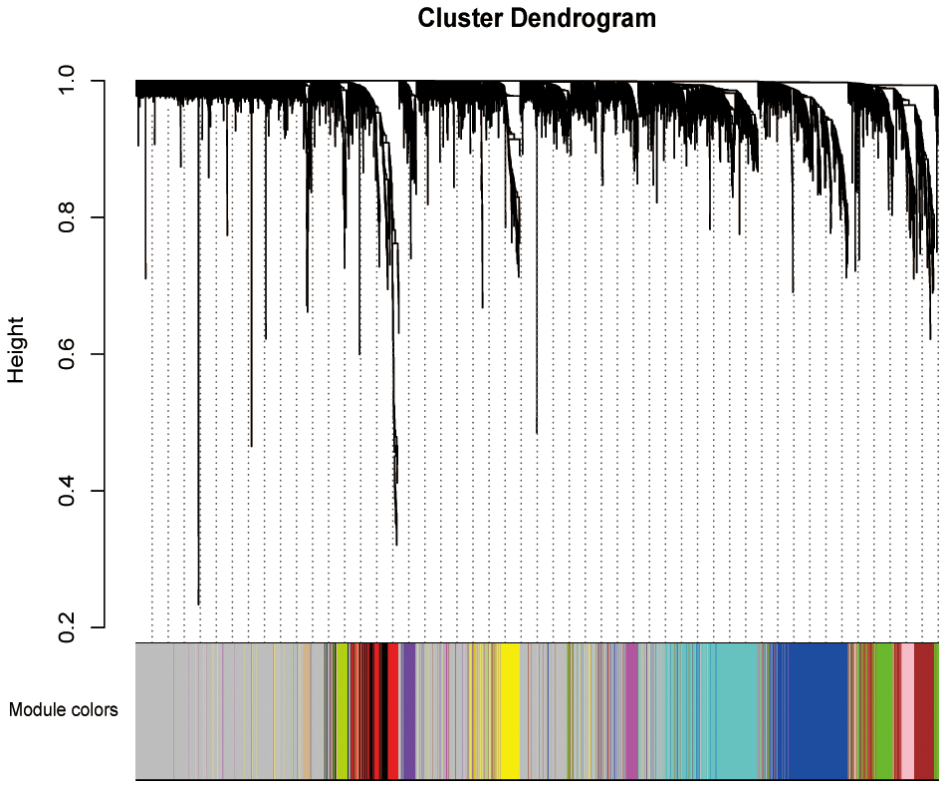
**Weighted co-expression network establishment.** The cluster dendrogram of the DEGs; each branch represents one gene and the co-expression modules are represented by different colors. DEGs, differentially expression genes.

4, 777 DEGs obtained and 176 PAAD samples with the information of pathological stage and grade were enrolled to construct a co-expression network. We then constructed phylogenetic tree and removed outliers, finally retained 162 samples. After assessing the quality of expression matrix of TCGA and GTEx, “WGCNA” package was used in R. The value of β = 9 was chosen to ensure the scale-free network. The MEDissThres was set as 5 to merge similar modules and finally 12 modules were obtained (Figure 2). Black module included 127 genes, blue module included 553 genes, brown module included 131 genes, green module included 215 genes, green-yellow module included 58 genes, grey module included 2142 genes, magenta module included 106 genes, pink module included 123 genes, purple module included 89 genes, red module included 177 genes, tan module included 42 genes, turquoise module included 562 genes, and yellow module included 249 genes. All DEGs of every module were listed in Supplementary Table 1. Genes contained in grey module were identified as not co-expressed, thus, these genes of grey module were deleted in the further analyses.

### Relationship among modules and key module identification

**Figure 3:**
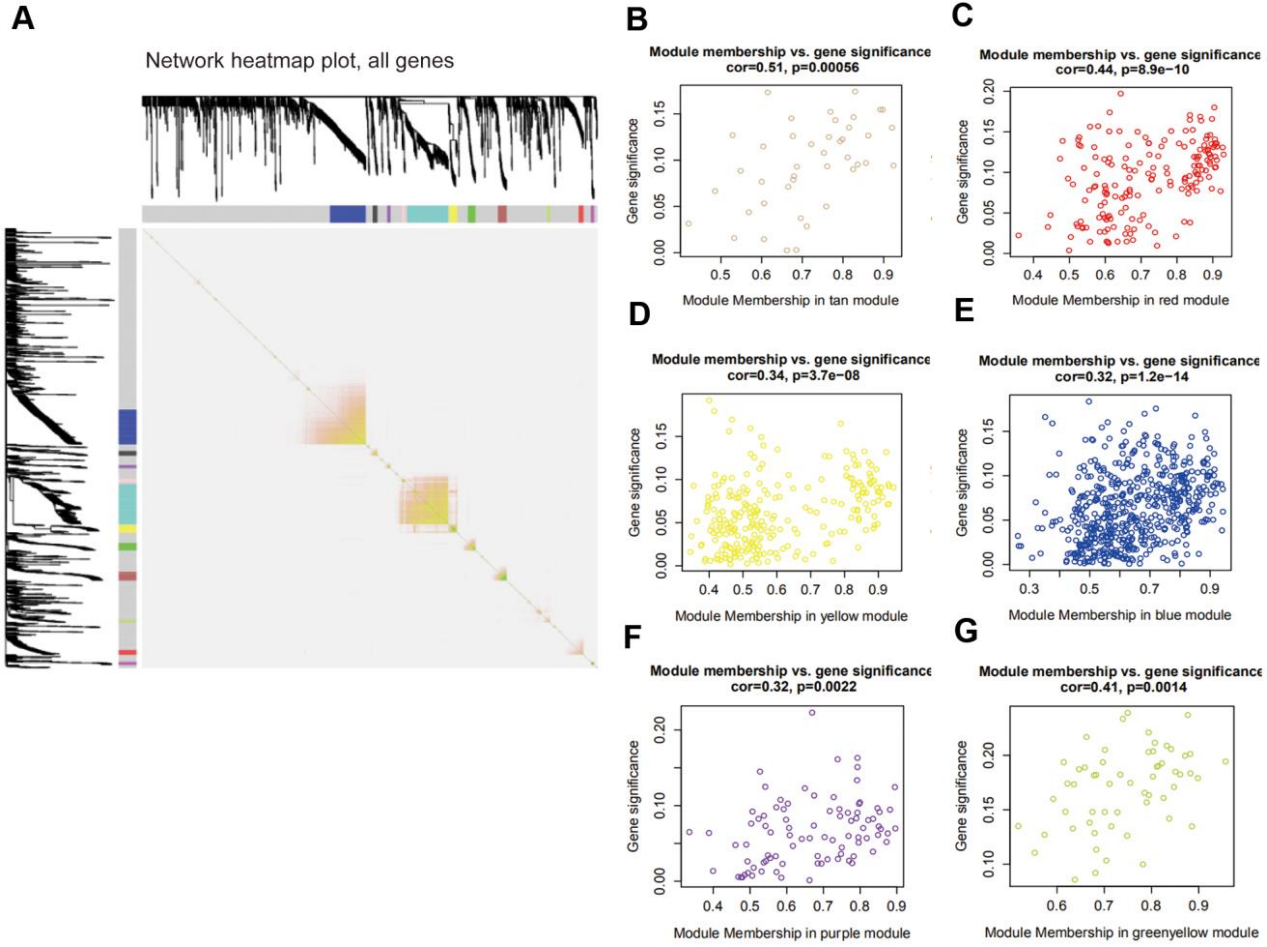
**Interaction among modules and identification of pivotal modules.** (**A**) Interaction of co-expression genes. Different colors of horizontal axis and vertical axis represent different modules. The brightness of yellow in the middle represents the degree of connectivity of different modules. (**B**–**G**) Scatter plots of module eigengenes in corresponding modules that correlated with clinical features.

The interaction of 12 modules was analyzed by the network heatmap (Figure 3A). This analysis revealed that every module was independent of each other, suggesting that these 12 modules posed high-scale independence degree and significant independence of genes expression in every module. Afterwards, correlation analysis was implemented between each module and clinical features. Results indicated that tan and red modules were significantly correlated with the tumor Nude (N) stage of PAAD (Figure 3B, 3C); yellow, blue and purple modules were remarkably associated with PAAD tumor Stage (S) stage (Figure 3D–3F); the one that related to Tumor (T) stage was green-yellow (Figure 3G).

### Enrichment analyses of blue module

**Figure 4:**
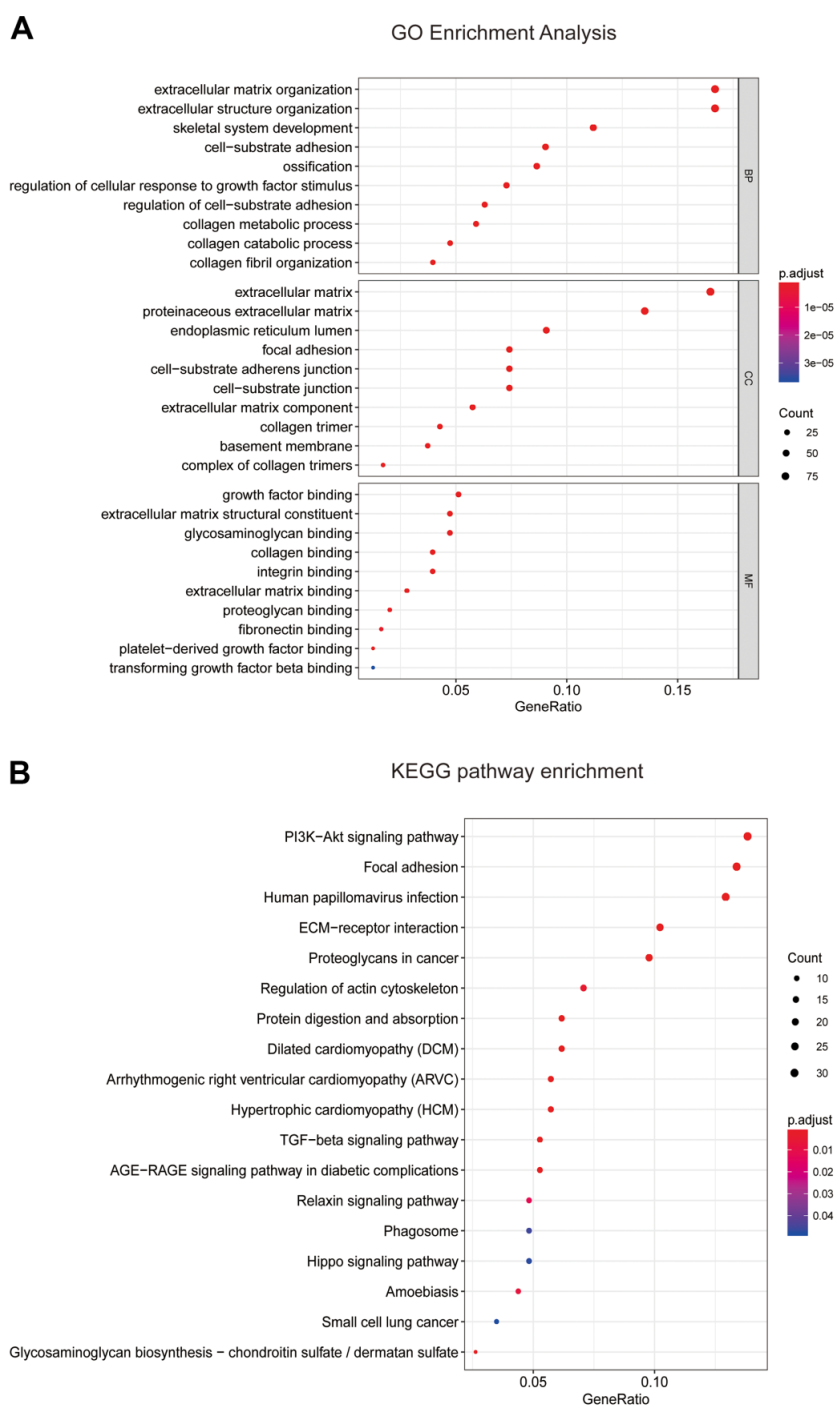
**Enrichment analyses of blue module.** (**A**) Go enrichment analysis and (**B**) KEGG pathway analysis of all genes in blue module. GO, Gene Ontology; KEGG, Kyoto Encyclopedia of Genes and Genomes.

On the basis of the correlation between identified modules and clinicopathological characteristics, we performed functional enrichment analyses to investigate the biological functions of DEGs that were included in the above mentioned modules (Figure 3B–3G). The results of enrichment analyses revealed that many important biological processes and pathways were enriched in blue module, while the gene enrichment results of other modules were not obvious. Thus, the blue module was selected as a key module for further analysis. In Figure 4A, GO analysis showed that DEGs of the blue module were mainly related with cell-substrate adhesion, cellular response to growth factor stimulus, epithelial to mesenchymal transition (EMT) and cell migration. Furthermore, KEGG enrichment analysis disclosed that these DEGs were significantly associated with ECM-receptor interaction, PI3K/Akt signaling pathway, TGF-β signaling pathway, Hippo signaling pathway and so on (Figure 4B).

### Key genes identification and validation

**Figure 5:**
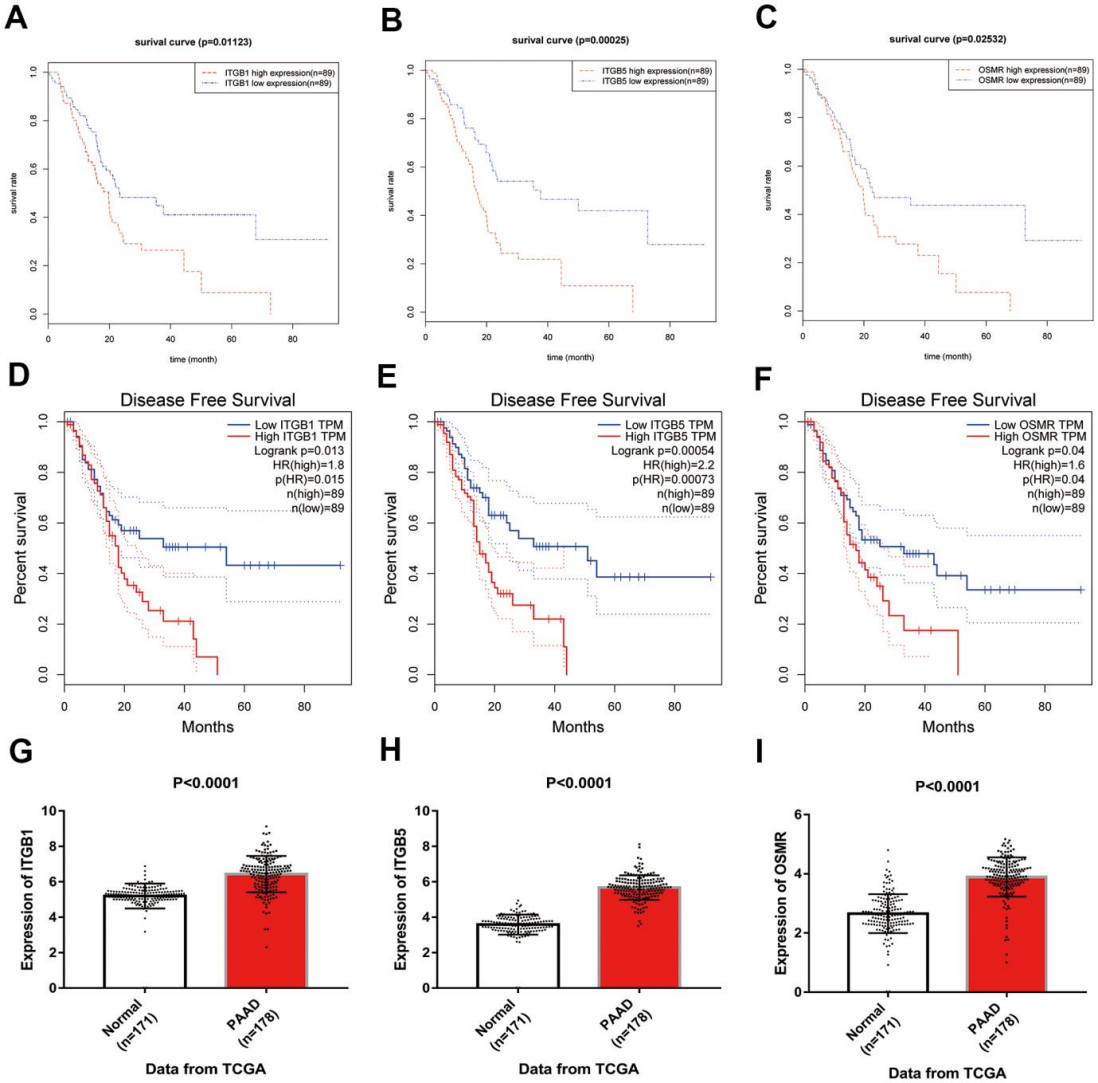
**Identification of key genes in PAAD.** (**A**–**C**) Overall survival curves of three key genes in PAAD. (**D**–**F**) Disease free survival analyses of three key genes in PAAD. (**G**–**I**) Expression levels of ITGB1, ITGB5, and OSMR in PAAD tissues and normal tissue samples. PAAD, pancreatic adenocarcinoma.

**Figure 6:**
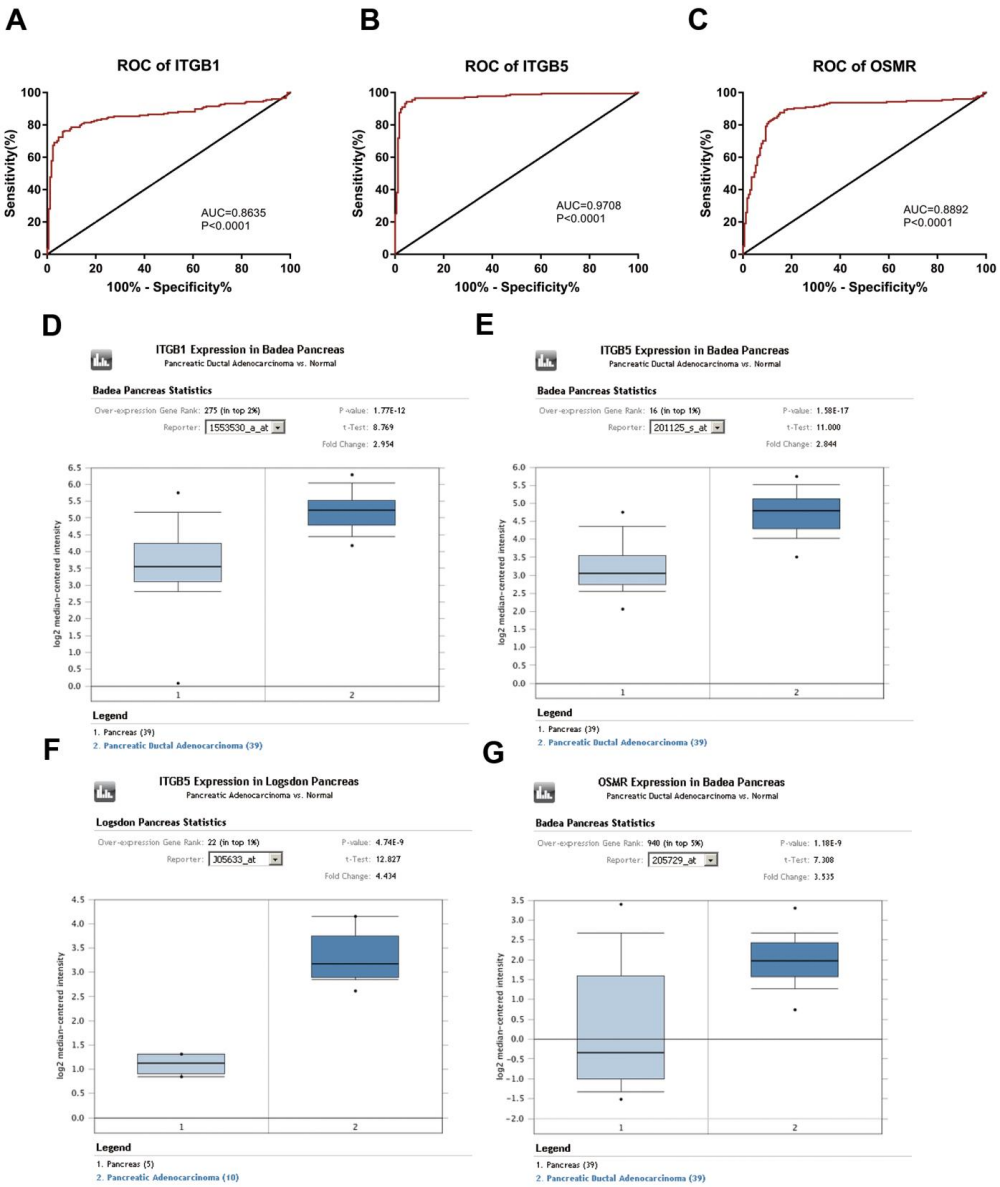
**Diagnostic significance of three key genes and validation of the expression levels of key genes.** (**A**–**C**) ROC curves and AUC were utilized to evaluate the specificity and sensitivity of key genes on PAAD diagnosis. (**D**–**G**) Data from Oncomine datasets confirmed that ITGB1, ITGB5, and OSMR were significantly increased in PAAD tissues compared with normal controls. ROC, receiver operating characteristic; AUC, area under the curve; PAAD, pancreatic adenocarcinoma.

Based on the results of KEGG enrichment analysis, we found that the majority genes of blue module were enriched in PI3K/Akt signaling pathway (Figure 4B). To identify the hub genes, Kaplan-Meier method was performed to plot the survival curves of genes enriched in PI3K/Akt pathway for the assessment of prognostic significance and three genes (*ITGB1*, *ITGB5*, and *OSMR*) were observed to be significantly associated with prognosis of PAAD patients. The detailed information of clinical samples were shown in Supplementary Table 3, and datasets used for survival analysis were exhibited in Supplementary Table 4. As shown in Figure 5A–5C, PAAD patients with high expression of ITGB1, ITGB5, and OSMR had poorer outcomes, while patients in down-regulation of ITGB1, ITGB5, and OSMR group showed a higher survival rate (P < 0.05). Similarly, ITGB1, ITGB5, and OSMR were also negatively associated with the disease free survival of PAAD patients (Figure 5D–5F, P < 0.05). The expression levels of ITGB1, ITGB5, and OSMR were remarkably increased in PAAD tissue samples in contrast to normal pancreas specimens (Figure 5G–5I, P < 0.0001). In addition, ROC curves revealed that ITGB1, ITGB5, and OSMR function as crucial factors on the diagnosis of PAAD (Figure 6A–6C, P < 0.0001). Afterwards, the Oncomine datasets were utilized to verify the expression levels of these three key genes and discovered that compared with the pancreas tissues, the PAAD tumor tissues exhibited high expression levels of ITGB1, ITGB5, and OSMR (Figure 6D–6G, P < 0.05). A prognostic model using OSMR, ITGB1 and ITGB5 was also constructed. The risk score distribution of three-gene signature was revealed in Supplementary Figure 1. Compared with other signature risk score that identified in previous reports [10–12], the predictive effect of our model on the 5-year survival rate was better than other models (Supplementary Figure 2). The expression levels of ITGB1, ITGB5 and OSMR in different stages were determined and showed in Supplementary Figure 3, which unveiled that the expression levels of ITGB1, ITGB5 and OSMR in the late stage were generally higher than those in the early stage. This result manifested that the three hub genes were not suitable as early diagnostic indicators, but of certain significance for the assessment of the progression and prognosis of PAAD. Prognostic values of ITGB1, ITGB5 and OSMR were tested using multivariate analyses, which exhibited in Supplementary Figure 4. Collectively, these observations elucidated that ITGB1, ITGB5, and OSMR might serve as important biomarkers for the prognosis and diagnosis of PAAD.

### miR-16 directly targets ITGB1 and ITGB5

**Figure 7:**
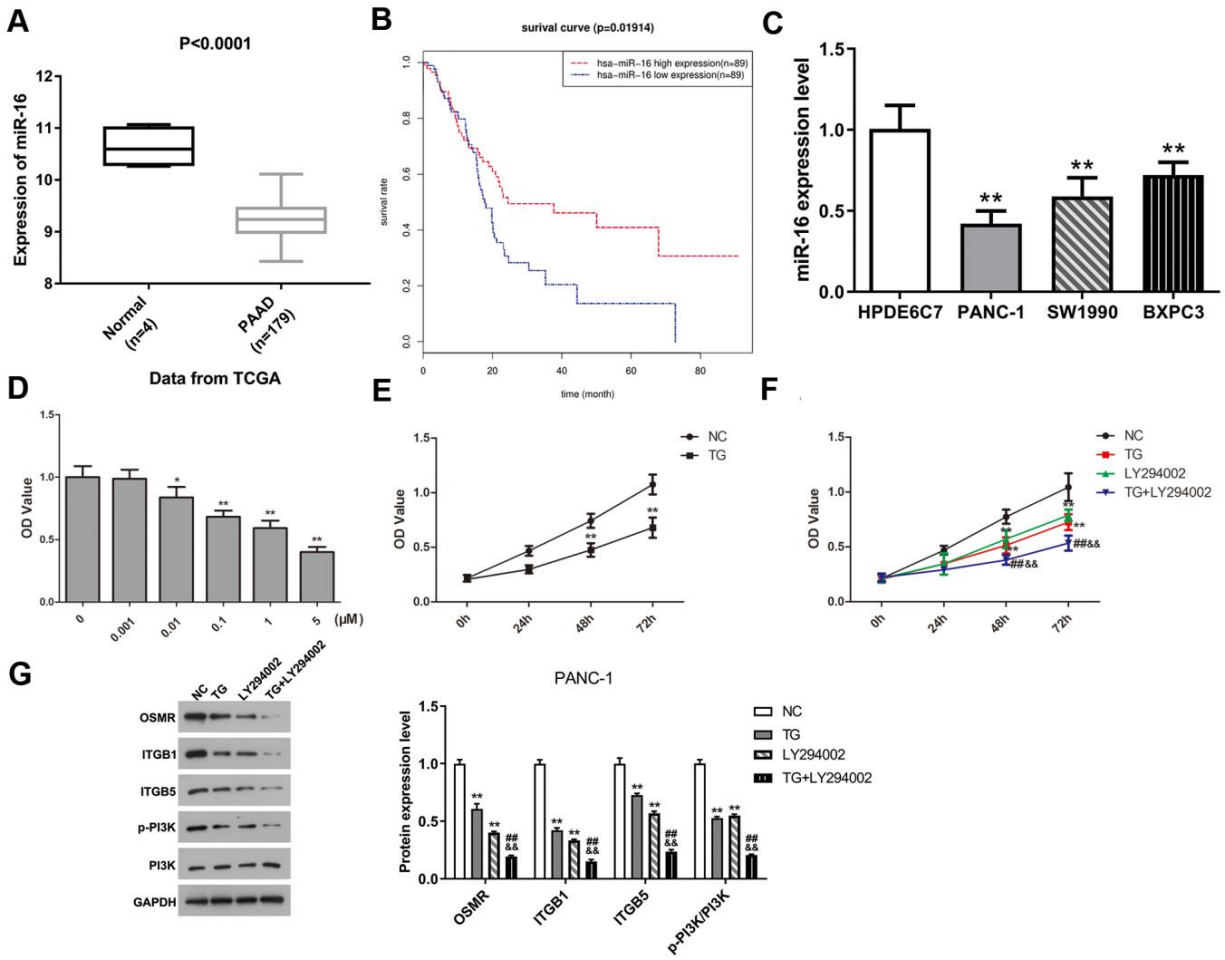
**MiR-16 expression was dramatically decreased in PAAD and positively correlated with prognosis of PAAD patients.** TG inhibited PANC-1 cell viability via modulating PI3K pathway. (**A**) Expression level of miR-16 between PAAD tissue samples and normal pancreatic tissues. (**B**) Overall survival curve of miR-16 in PAAD patients was plotted using Kaplan-Meier analysis. (**C**) The expression level of miR-16 in HPDE6C7, PANC-1, SW1990, and BXPC3. (**D**, **E**) Cell proliferative ability was measured using CCK-8 assay after TG treatment with different concentrations or times. (**F**) Cell viability was detected followed by TG or LY294002 induction. (**G**) Western blot was performed to examine the expression of OSMR, ITGB1, ITGB5, p-PI3K and PI3K in PANC-1 cells. PAAD, pancreatic adenocarcinoma; TG, thapsigargin.

To explore the potential molecular mechanism of three hub genes, we firstly screened the DEmiRs in PAAD based on the TCGA database. The limma package of R software was applied to conduct difference analysis in accordance with the threshold (|logFC| > 1 and FDR < 0.05). A total of 17 DEmiRs were obtained, including 2 up-regulated miRs and 15 down-regulated miRs. The miRs that have potential interactions with ITGB1, ITGB5, and OSMR were predicted using TargetScan website. The predicted miRs of three hub genes and DEmiRs that were decreased in PAAD were intersected, and results showed that ITGB1 and ITGB5 had a common regulatory miR, namely miR-16. Therefore, we speculated that miR-16 might inhibit the progression of PAAD by mediating the ITGB1/ITGB5/PI3K/Akt signaling pathway. To further validate the hypothesis, we subsequently analyzed the expression level of miR-16 in PAAD tissues and found that miR-16 expression was significantly reduced in PAAD tissue samples compared with normal controls (Figure 7A, P < 0.0001). Figure 7B suggested that miR-16 was positively correlated with survival rates of PAAD patients (P < 0.05). Moreover, miR-16 was expressed in PAAD cell lines at lower levels relative to HPDE6C7 (Figure 7C, P < 0.01). PANC-1 cell line was selected in the following experiments due to its lowest miR-16 expression. In summary, these findings demonstrated that miR-16 might affect the development of PAAD via regulating ITGB1/ITGB5.

### Screening of related small molecule drugs

In order to screen out small molecule drugs candidates of PAAD, CMap was implemented on the basis of the analysis of consistent differently expressed probesets between PAAD tumor samples and normal specimens. The 29 small molecule drugs with highly significant correlations were filtered as the criterion of P < 0.01 and listed in Table 1. Among these small molecule drugs, thapsigargin (TG), viomycin, adiphenine, and CP-690334-01 revealed higher negative correlations and potential to reverse the PAAD tumor status.

**Table 1:** Results of CMap analysis.

**cmap name**	**mean**	**n**	**enrichment**	**p**	**specificity**	**percent non-null**
**thapsigargin**	-0.857	3	-0.99	0	0.0065	100
trichostatin A	0.291	182	0.349	0	0.5924	51
8-azaguanine	0.75	4	0.891	0.00014	0.0296	100
**viomycin**	-0.633	4	-0.87	0.00058	0.0504	100
mepacrine	0.792	2	0.979	0.00074	0.0052	100
PHA-00745360	-0.272	8	-0.649	0.00086	0.0078	50
thiamphenicol	-0.435	5	-0.781	0.0009	0.0333	80
emetine	-0.495	4	-0.851	0.00093	0.0588	100
Prestwick-692	-0.567	4	-0.845	0.00103	0.0068	100
heptaminol	-0.32	5	-0.773	0.00106	0.0137	60
proscillaridin	0.745	3	0.92	0.00112	0.0545	100
genistein	-0.351	17	-0.454	0.00114	0.0929	58
bisacodyl	0.681	4	0.826	0.00143	0.0053	100
indoprofen	-0.488	4	-0.828	0.00165	0.02	100
cephaeline	-0.498	5	-0.75	0.00182	0.1325	80
monensin	-0.483	6	-0.693	0.00201	0.0894	83
etiocholanolone	-0.306	6	-0.689	0.00219	0.0779	66
pargyline	0.574	4	0.815	0.00223	0.0158	100
bepridil	0.554	4	0.812	0.00237	0.0389	100
felbinac	-0.33	4	-0.81	0.00257	0.0426	75
**adiphenine**	-0.601	5	-0.734	0.00274	0.1613	80
canadine	-0.319	4	-0.8	0.0031	0.0201	50
trazodone	0.706	3	0.878	0.00349	0.0373	100
6-bromoindirubin-3'-oxime	-0.383	7	-0.626	0.00355	0.0794	71
nadolol	-0.34	4	-0.783	0.00442	0.0769	50
valproic acid	0.235	57	0.229	0.00447	0.1908	54
albendazole	0.655	3	0.851	0.00615	0.0058	100
pheneticillin	-0.349	4	-0.759	0.00692	0.0131	75
**CP-690334-01**	-0.436	8	-0.552	0.00794	0.0816	62

P < 0.01. Bold fonts represent small molecule drugs with higher negative correlation and the potential to treat pancreatic adenocarcinoma (PAAD).

### TG treatment inhibits PANC-1 cells viability through mediating PI3K/Akt pathway

To assess the potential role of TG in PAAD, we performed CCK-8 test to detect the proliferative capability of PANC-1 cells after TG treatment. The proliferation of PANC-1 cells was inhibited in a dose-/time-dependent manner (Figure 7D, 7E, P < 0.05). PANC-1 cell viability was suppressed attributed to the treatment TG (1 μM), LY294002 or both (Figure 7F, P < 0.01). Moreover, the expression of OSMR, ITGB1, ITGB5, and p-PI3K were all decreased after TG (1 μM) treatment in PANC-1 cells (Figure 7G, P < 0.01). LY294002 stimulation induced a consistent expression tendency (Figure 7G, P < 0.01). The combination of TG and LY294002 further reduced the expression of OSMR, ITGB1, ITGB5, and p-PI3K (Figure 7G, P < 0.01). Western blot assay further verified the inhibitory effect of TG on the PI3K/Akt pathway (Supplementary Figure 5, P < 0.01). These results showed that TG might exert an inhibitory role through downregulating OSMR, ITGB1, and ITGB5 via mediating the PI3K/Akt signaling pathway in PAAD.

## DISCUSSION

Recently, relying on the widespread of novel chemotherapeutic drugs and progress in surgery, the PAAD treatment has partial improved. However, most of PAAD patients are diagnosed with advanced stage, thus, the survival rate of PAAD patients still remain low [13, 14]. Therefore, it is urgently needed to identify the reliable biomarkers for the exact diagnosis of PAAD. In the present exploration, we conducted comprehensive bioinformatics analyses and identified three key genes as well as several potential small molecule drugs to treat PAAD.

Three hub genes OSMR, ITGB1, and ITGB5 might promote the progression of PAAD, and high expressions of them were all associated with poor prognoses in PAAD patients. Up to date, biological role of these three genes has not been detected in PAAD. *OSMR* gene is located in chromosome 5p13.1 and encodes an important protein OSMRβ, which can be heterodimerized with IL-6 and form the type II OSMR [15]. It has been reported that type II OSMR is correlated with cell viability, differentiation, inflammatory responses and metastasis [16]. Association between the polymorphism of OSMR gene and bladder cancer has been determined, which reveals that OSMR could affect the recurrence rate, overall survival and tumor grade [17]. Xu et al. suggest that OSMR is a prognostic biomarker in glioblastoma and overexpression of OSMR could affect the immune response within the progression of glioblastoma [18, 19]. ITGB1 is considered as a key adhesion receptor for ECM components and could contribute to cell proliferation, invasion and metastasis in various cancers [20, 21]. Up-regulation of ITGB1 can stimulate the proliferation, invasion and migration capacities of colorectal cancer cells [22]. ITGB1 is crucial for fascin-mediated breast cancer stem cell function and disease progression [23]. Lin et al. demonstrated that miR-185/ITGB5 plays an essential role in HCC through mediating β-catenin pathway [24]. The tumorigenic ability of breast cancer cells would be strengthened by the ITGB5-mediated EMT [25]. More importantly, Wortzel et al. highlight that ITGB5 is enriched in the exosomes of liver metastatic PAAD [26]. To further validate the significance of three hub genes, ROC analysis showed that ITGB1, ITGB5, and OSMR had significant diagnostic potentials in PAAD, and all were remarkably increased in PAAD tissue samples compared with normal controls. These findings confirmed that ITGB1, ITGB5, and OSMR might serve as important prognostic and diagnostic biomarkers in PAAD.

In order to explore how three key genes regulate the development of PAAD, we screened out the DEmiRs in PAAD based on TCGA database, and ultimately obtained 2 up-regulated miRs and 15 down-regulated miRs. Then, integrated the above miRs with the upstream miRs of ITGB1, ITGB5, and OSMR, miR-16 was selected due to it is the common regulator of ITGB1 and ITGB5. Previous researches have indicated that miR-16 is involved in a variety of tumors, including cervical cancer [27], bladder cancer [28], and lung cancer [29]. Our results illustrated that miR-16 was significantly decreased in PAAD tissues and PAAD patients with low miR-16 expression had poor outcomes. Additionally, three hub genes were enriched in PI3K-Akt pathway. ITGB1, targeted by specific miRNAs, could affect the activity of PI3K-Akt pathway in several tumors, such as breast cancer [30], cervical cancer [31] and hepatocellular carcinoma [32]. LINC00520 sponging miR-27b-3p regulates OSMR expression to stimulate acute kidney injury via mediating the PI3K-Akt pathway [33]. Therefore, we speculated that miR-16 might inhibit the invasion and metastasis of PAAD through regulating ITGB1/ITGB5-mediated PI3K-Akt pathway. Furthermore, miR-16 has been verified as a potential diagnostic target involved in the development of PAAD [34]. Therefore, further investigation of miR-16/ITGB1/ITGB5 axis in PAAD may be of great importance.

CMap is a useful tool for identifying the correlation between small molecule drugs and diseases based on the gene expression profiles [35]. To determine small molecule drugs with potential roles to treat PAAD, we conducted CMap analysis and observed that TG, viomycin, adiphenine, and CP-690334-01 may have potential therapeutic efficacy against PAAD. Of which, TG and adiphenine have been proven to possess anti-cancer effects while the role of the other two small drugs has not been studied. TG is an endoplasmic-reticulum Ca(^2+^)-ATPase pump inhibitor and correlated with the activation of NF-kappaB translocation in pancreatic cancer [36]. Moreover, TG can directly induce ER stress and decline the cell stemness [37]. The treatment of TG could inhibit cell viability via mediating PI3K pathway. Adiphenine is widely used in clinical practice of local anesthetics, and reported as an adjuvant small drug against ovarian cancer [38]. Therefore, we concluded that these identified small molecule drugs might have potential to treat PAAD.

However, there are some limitations in the present study. First, the effects of hub genes on the phenotype of PAAD are insufficient and the possible molecular mechanism also needs to be further verified in the future. Second, this was only a retrospective investigation based on the public available databases, further *in vivo* and *in vitro* experiments should be performed to confirm the current results.

## CONCLUSIONS

In summary, the present study identified three hub genes that likely promote the development of PAAD, which may function as novel therapeutic targets and contribute to guide the selection of individual therapies in PAAD.

## MATERIALS AND METHODS

### Data collection of gene expression profiles

To identify the DEGs in PAAD, the RNA-Seq data of PAAD were downloaded from the TCGA database (https://portal.gdc.cancer.gov/), which contains 178 tumor samples and 4 normal samples. Due to there were only 4 normal samples of adjacent tissue samples, we integrated the RNA-Seq data of 171 normal pancreas specimens from the Genotype-Tissue Expression (GTEx) dataset (https://gtexportal.org/) with 4 normal samples as control group. All data retrieved from TCGA and GTEx were corrected and standardized using normalizeBetween Arrays function of the limma package in R (Supplementary Table 2).

### Data pre-processing and DEGs identification

The “limma” package of R software was used to standardize and transform the RNA-Seq data for the DEGs identification in PAAD [39]. |logFC (fold change)| > 1 and false discovery rate (FDR) < 0.05 were considered as the threshold for DEGs screening.

### Weighted co-expression network analysis

As previously mentioned, a co-expression network can be established by “WGCNA” R package [40]. The input data of WGCNA establishment consists of 4777 DEGs that we had obtained and 176 PAAD samples with pathological staging. At first, clustering the samples and selecting a soft threshold value β based on the standard scale-free networks. Second, the adjacencies among all filtered genes were determined using the power adjacent function to Pearson correlation matrix and then transformed these data into topological overlap matrixs (TOMs). Average linkage hierarchical clustering was performed after calculating the dissimilarity (1-TOM). Finally, in order to further analyze the module, the dissimilarity of module eigengenes was calculated and modules with high similarity were merged together following the cut-off value of 5.

### Identification of significant clinical modules

Pearson’s correlation analysis was used to determine the relevance between clinical features and modules. The module membership (MM) represents the correlation coefficient between genes and module eigengene (ME) that refers to first principal component of module and the expressional pattern of genes, which was utilized to describe the association between a gene and its belonging to the module. Gene significance (GS) represents a linear relationship between gene expression and clinical phenotypes. Genes with high GS and MM were identified as hub genes in a module.

### KEGG and GO enrichment analyses

The biological functions of genes in blue module were predicted using GO and KEGG enrichment analyses based on the on-line platform of Database for Annotation, Visualization, and Integrated Discovery (DAVID) (http://david.abcc.ncifcrf.gov/). Adjusted P < 0.05 was regarded as the cut-off criteria.

### Validation of key genes and risk score signature construction

To further investigate the possible roles of module genes in the incidence and progression of PAAD, we conducted Kaplan-Meier analysis on the genes of PI3K/Akt signaling pathway with the most abundant genes in blue module to screen the key genes that have significant prognostic significance in PAAD. The clinical specimens were collected from the TCGA database. The receiver operating characteristic (ROC) curve was plotted to compare the sensitivity and specificity of key genes we obtained. The Oncomine datasets were utilized to verify the significant difference of hub genes. The Cox regression analysis was utilized to establish the prognostic model, following the formula: risk score = expmRNA1*betamRNA1+…+expmRNAn*betamRNAn. The “exp” denotes the standardized expression of each identified genes, and “beta” value was determined using multivariate Cox regression analysis.

### Analysis and identification of DEmiRs

Identification of DEmiRs was performed using the miR-Seq expression profiles based on TCGA database (a total of 182 samples, including 178 tumor samples and 4 normal specimens). The specific steps were the same as the screening of DEGs, as above described. TargetScan website was used to predict the miRs that target key genes.

### Identification of potential small molecules

The Connectivity Map (CMap, http://www.broadinstitute.org/cmap/) was employed to screen the potential small molecule drugs that were closely correlated with indicated disease [41]. DEGs that we identified were contrasted with those genes related with the small molecule drugs treatment in CMap database to screen the potential small molecule drugs linked with DEGs. The range of connectivity value is from -1 to 1: the positive value represents that the small drug can activate the PAAD cells, while negative value represents that these small molecules can be used to reverse the status of PAAD cells.

### Cell culture

Human PAAD cell lines PANC-1, SW19901, BXPC3, and normal pancreatic cells (HPDE6C7) were purchased from the cell bank of Chinese Academy of Science (Shanghai, China). Cells were incubated in DMEM medium (Gibco, Carlsbad, CA, USA) supplemented with 10 % FBS and 1 % penicillin-streptomycin (Gibco, Carlsbad, CA, USA) at 37° C in a humidified atmosphere with 5 % CO_2_. To measure the biological role of TG in cells, TG was obtained from Shanghai Amquar Ltd. (Shanghai, China) and dissolved in DMSO (Solarbio, Beijing, China) for further experiments. LY294002, a PI3K inhibitor, was purchased from MedChemExpress (Monmouth Junction, NJ, USA) and utilized to treat cells at a concentration of 10 μM.

### Cell counting kit 8 (CCK-8) assay

TG was diluted into DMEM to concentrations of 0, 0.001, 0.01, 0.1, 1 and 5 μM. Cells were cultured with TG for 48 h and their proliferative ability were assessed using CCK-8 kit (Dojindo Molecular Technologies, Inc., Kumamoto, Japan). The absorbance of cells at 450 nm was detected under the Elx800 Reader (Bio-Tek Instruments, VT, USA).

### Western blot

After specific treatment, proteins of cells were lysed by RIPA buffer (Solarbio, Beijing, China). The concentration of separated protein was measured with a BCA Kit (Sangon Biotech, Shanghai, China). Followed by loaded in 10 % SDS-PAGE, proteins were transferred onto PVDF membranes (Millipore, Danvers, MA). Afterwards, PVDF membranes were blocked with 5 % skimmed milk, and incubated with anti-OSMR, anti-ITGB1, anti-ITGB5, anti-p-PI3K, anti-PI3K, anti-p-AKT, anti-AKT, anti-p-mTOR, anti-mTOR and GAPDH antibodies at 4° C overnight. The secondary antibody was used to incubate membranes at room temperature for 1 h after rising. All antibodies were purchased from Cell Signaling Technologies (Danvers, MA, USA). The protein bands were probed with enhanced chemiluminescence (ECL, Millipore, USA) and captured with an ImageJ software (National Institutes of Health, MD, USA).

### Data availability statement

The datasets generated and/or analysed during the present study are available from the corresponding author upon reasonable request.

## Supplementary Material

Supplementary Figures

Supplementary Table 1

Supplementary Table 2

Supplementary Table 3

Supplementary Table 4
