# Supplementary material for: Identification of novel candidate biomarkers for pancreatic adenocarcinoma based on TCGA cohort
Source: Aging (Albany NY). 2021 Feb 11;13(4):5698–717. doi: 10.18632/aging.202494 (PMC7950294; doi:10.18632/aging.202494)
Supplement: Supplementary Figures [file aging-13-202494-s001.pdf]

SUPPLEMENTARY FIGURES

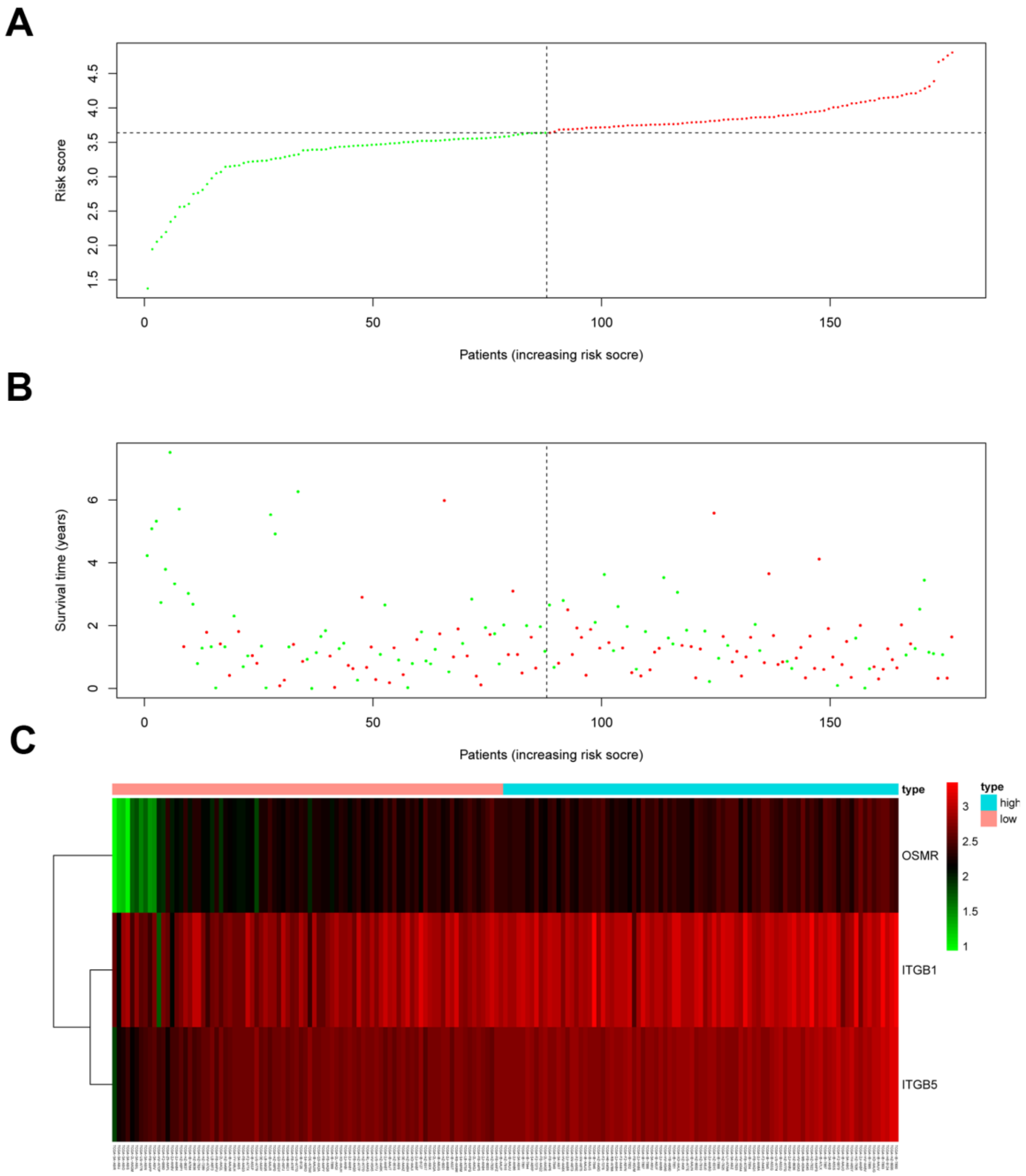

Supplementary Figure 1. Risk score analysis, survival status and survival time between two risk group and expression distribution of the three-gene signature. (**A**) The three-gene signature risk score distribution. (**B**) Scatterplot of patients with PAAD survival status ordered by risk score. (**C**) The heat-map of the three-gene expression files. PAAD, pancreatic adenocarcinoma.

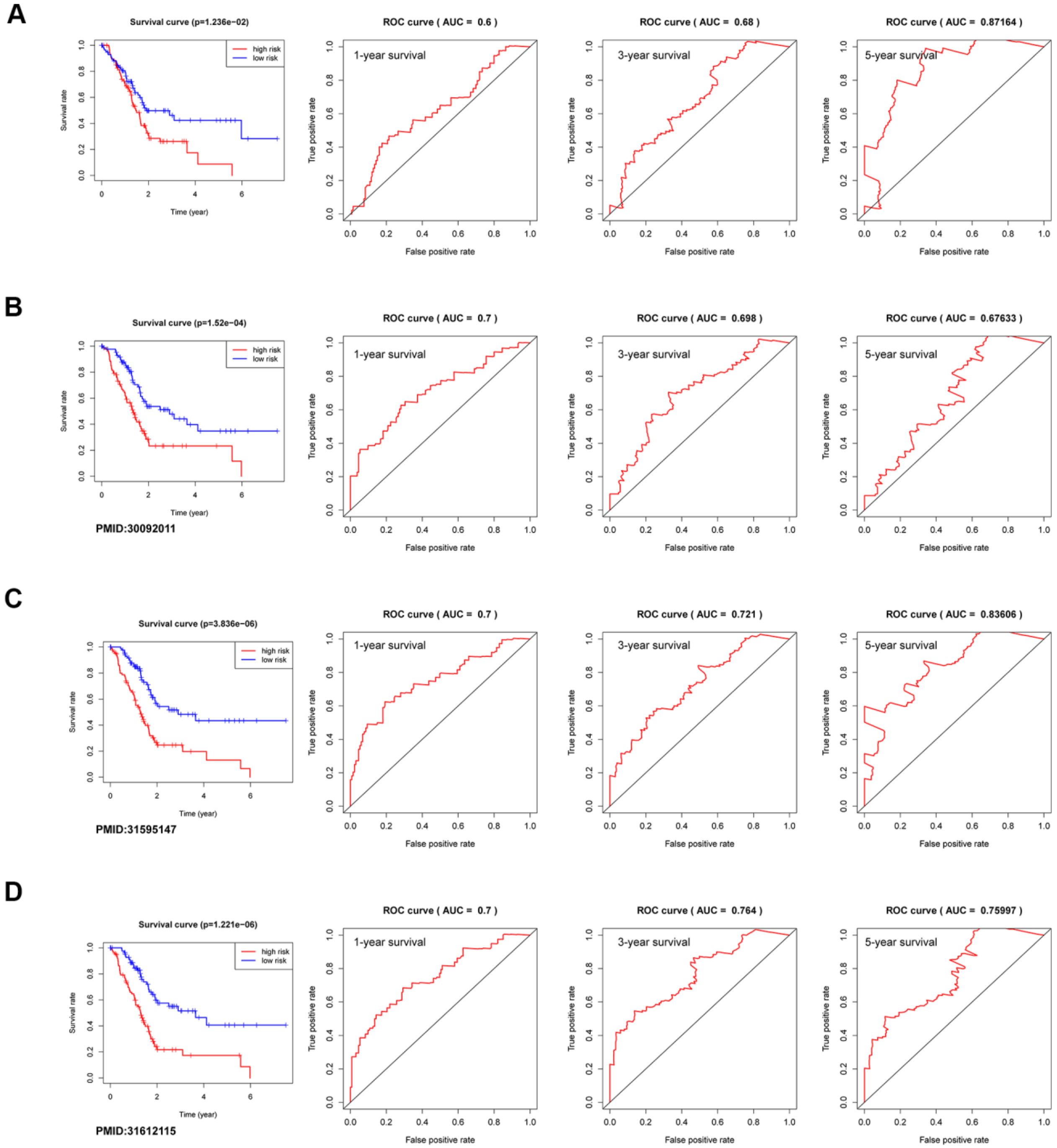

Supplementary Figure 2. Comparison between three-gene signature and other signatures determined in previous investigations. (**A**–**D**) The overall survival and 1-year, 3-year and 5-year survivals of this risk score signature.

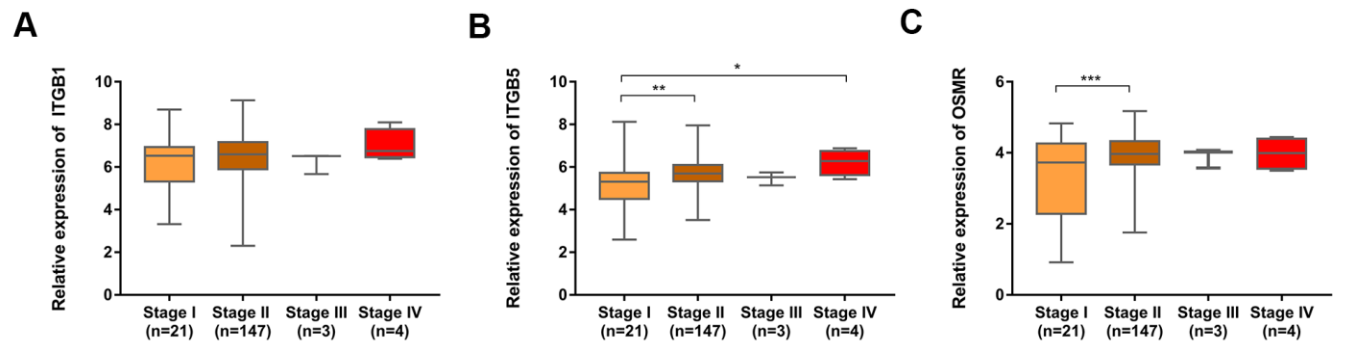

Supplementary Figure 3. The expression levels of ITGB1, ITGB5 and OSMR in the late stage were generally higher than those in the early stage. (**A**) The expression of ITGB1, (**B**) ITGB5, and (**C**) OSMR in different stages based on TCGA database.

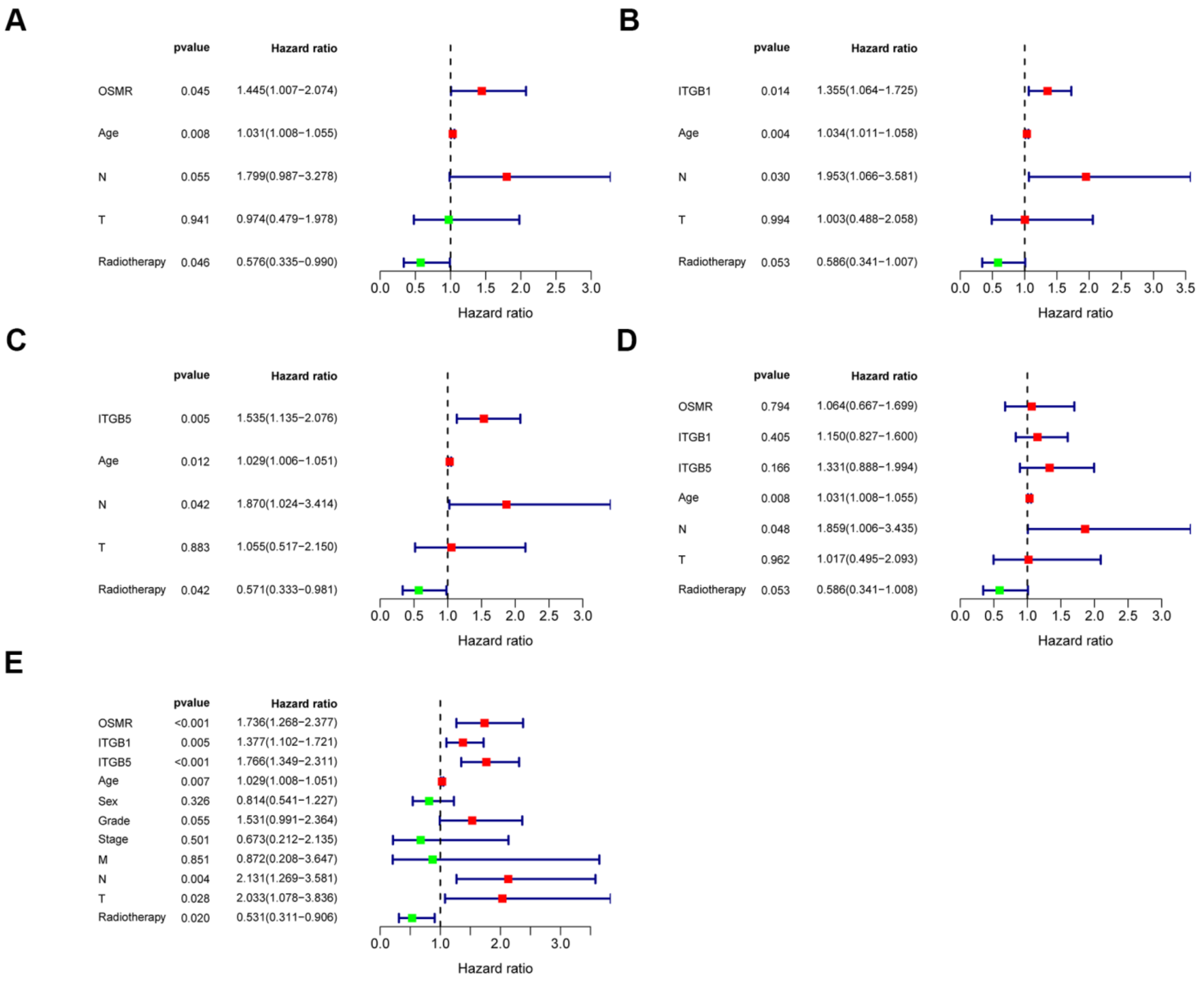

Supplementary Figure 4. Prognostic values of the three genes (ITGB1, ITGB5, and OSMR) tested by univariate and multivariate analyses. (**A**–**D**) Multivariate analysis. (**E**) Univariate analysis.

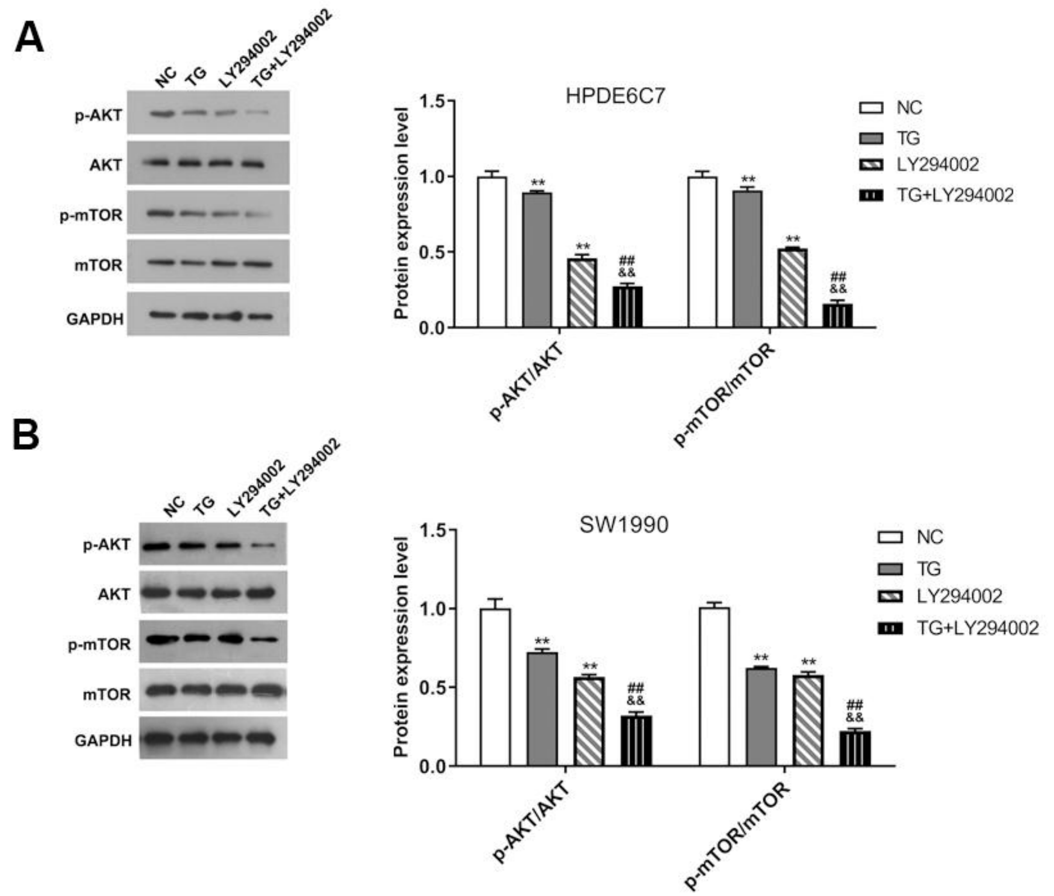

Supplementary Figure 5. TG inactivates the PI3K/AKT pathway in PAAD cells. The expression levels of p-AKT, AKT, p-mTOR and mTOR were detected using western blot in (**A**) HPDE6C7 and (**B**) SW1990 cells. PAAD, pancreatic adenocarcinoma; TG, thapsigargin.
