# Supplementary material for: Identification of novel candidate biomarkers for pancreatic adenocarcinoma based on TCGA cohort
Source: Aging (Albany NY). 2021 Feb 11;13(4):5698–717. doi: 10.18632/aging.202494 (PMC7950294; doi:10.18632/aging.202494)
Supplement: Supplementary Table 3 [file aging-13-202494-s004.pdf]

Supplementary Table 3. Detailed information of 162 clinical samples to be used for the construction of WCGNA.

Variables	Number
**Grade**	
G1+G2	113 (70%)
G3+G4	49 (30%)
**Stage**	
Stage I+II	154 (95%)
Stage III+IV	8(5%)
**T-stage**	
T1+T2	25(15%)
T3+T4	137(85%)

Note: T, tumor.
